# The Functional Neurological Disorder special collection in *Brain Communications*: bringing FND into the mainstream

**DOI:** 10.1093/braincomms/fcaf481

**Published:** 2026-01-08

**Authors:** Jon Stone

**Affiliations:** Centre for Clinical Brain Sciences, University of Edinburgh, Edinburgh EH16 4SA, UK; Department of Clinical Neurosciences, Royal Infirmary of Edinburgh, Edinburgh EH16 4SA, UK

## Abstract

Our Guest Editor, Jon Stone, introduces a special collection of articles focusing on functional neurological disorder.

The first time I ever presented an abstract at a scientific meeting on functional neurological disorder (FND), at the Association of British Neurologists meeting in 2001, it was inevitably in the ‘miscellaneous’ section of the meeting.^1^ At the time, FND was invisible in textbooks and neurology curricula, even though the condition was as common as it is now. There was so little research; one colleague expressed surprise that research on FND was even possible.

It is therefore a pleasure to be asked to introduce the *Brain Communications* special collection for FND. It is another marker of how much of an inroad this disorder has made into mainstream neurological practice and research in the last 25 years. The purpose of the special collection is to signal that *Brain Communications* welcomes submissions spanning both clinical and neuroscience research on FND and to allow readers to find published articles gathered in one location.

The breadth of the articles in the collection demonstrates the momentum of the field and its growing maturity. In the late 1990s, it was a diagnosis of exclusion, framed almost exclusively within a psychiatric paradigm, in which anecdote and speculation were dominant. In contrast, this collection reflects the diversity and rigour of modern scientific FND research. There are papers on diagnostic and classification issues, for example, revisiting Briquet’s syndrome of multiple functional symptoms and disorders,^2^ the clinical and mechanistic overlap of migraine with FND^3^ and whether you can us univariate resting-state EEG to distinguish functional from epileptic seizures^4^ (spoiler alert—you can't). A second cluster of papers examines mechanistic questions in individuals with FND, such as the relationship between post-movement beta synchronization and hyperbinding between perception and action,^5^ the correlation between resting-state network integration and segregation and self-reported somatic symptoms^6^ and reduced respiratory sensitivity in FND,^7^ highlighting growing interest in interoception.

Studies of treatment of FND are also represented, including a feasibility trial of a digital intervention for functional cognitive disorder from our own group,^8^ as well as a treatment-oriented review examining the question of whether metacognition is really impaired in FND.^9^ Together, the papers in the collection show the range of scientific ambition now being applied to FND and the many diverse perspectives from psychiatry, neurology, epidemiology, neuroscience and others needed to properly grapple with the clinical phenomena that present so commonly to health services around the world.^10^

FND has become a genuinely interdisciplinary field. The international FND Society, founded in 2019, reflects this in its membership of psychologists, psychiatrists, neurologists, allied health professionals and many others relevant to clinical management and research. Despite such progress, historical neglect and stigma still affect millions of people with FND worldwide. Bringing it into the mainstream, especially through scientific research, is arguably the most effective way of overcoming that legacy. Inclusion in neurology training curricula in the UK^11^ and Europe,^12^ as well as scientific panels, and US consensus guidelines,^13^ all signal—like this special collection—that what was once a ‘miscellaneous’ oddity is now more widely recognized.

Finally, a gentle note of caution, as the field turns towards neuroimaging, computational modelling and biomarkers, it could be easy to lose sight of the centrality of clinical science in FND. The presence of an FND collection in *Brain Communications* is another milestone but should not be misinterpreted as an exclusively ‘neurocentric’ approach to the disorder. The brain is the organ of psychiatry too, and clinical, psychological and social studies are also important. FND is a disorder where scientific approaches from neurons to brains, from person to society, are all relevant and welcome.^14^
